# Concomitant Use of Antiplatelet Agents and Proton-Pump Inhibitors Increases the Risk of Adverse Cardiovascular Events: A Nationwide Population-Based Cohort Study Using Balanced Operational Definitions

**DOI:** 10.3390/jcdd10060264

**Published:** 2023-06-20

**Authors:** Hee Gyu Yang, Dong-Kyu Kim

**Affiliations:** 1 Department of Otorhinolaryngology-Head and Neck Surgery, Chuncheon Sacred Heart Hospital, Hallym University College of Medicine, Chuncheon 24252, Republic of Korea; gmlrb123@hallym.or.kr; 2 Division of Big Data and Artificial Intelligence, Institute of New Frontier Research, Hallym University College of Medicine, Chuncheon 24252, Republic of Korea

**Keywords:** proton-pump inhibitor, antiplatelet, cohort, cardiovascular

## Abstract

Antiplatelet agents are commonly used in combination with proton-pump inhibitors (PPIs) in patients with acute coronary syndrome who are at risk of gastrointestinal hemorrhage. However, studies have reported that PPIs can alter the pharmacokinetics of antiplatelet agents and result in adverse cardiovascular events. We enrolled 311 patients who received antiplatelet therapy with PPIs for >30 days and 1244 matched controls following a 1:4 propensity score matching during the index period. Patients were followed up until death, myocardial infarction, coronary revascularization, or the end of the follow-up period. Patients who used antiplatelet therapy with PPIs were found to be at higher risk of mortality than the controls (adjusted hazard ratio (HR): 1.77; 95% confidence interval (CI): 1.30–2.40). The adjusted HR for patients who used antiplatelet agents with PPIs developing myocardial infarction and coronary revascularization events was 3.52 (95% CI: 1.34–9.22) and 4.74 (95% CI: 2.03–11.05), respectively. Additionally, middle-aged patients or those within 3 years of concomitant use showed a higher risk of myocardial infarction and coronary revascularization. Our findings suggest that antiplatelet therapy combined with PPIs has a higher mortality risk in patients with gastrointestinal bleeding and is associated with an increased risk of myocardial infarction and coronary revascularization.

## 1. Introduction

In the pathogenesis of arterial thrombosis, platelets are responsible for the initiation of a series of complex interactions that culminate in platelet aggregation and thrombus formation. Therefore, an antiplatelet therapy such as aspirin and clopidogrel is the standard treatment for patients with acute coronary syndrome, particularly those undergoing percutaneous coronary intervention [1]. Antiplatelet therapy is also recommended for the secondary prevention of other vascular events in patients with strokes, transient ischemic attacks, or peripheral arterial disease [2]. During the last century, antiplatelet agents have significantly improved patient clinical outcomes owing to the prevention of a substantial number of atherothrombotic events, decreasing cardiovascular mortality rates. However, antiplatelet agents also have limited clinical use for potential adverse gastrointestinal (GI) complications, including peptic ulcers, GI bleeding, or perforations, especially in patients with previous GI events. An expert consensus report revealed that dual antiplatelet therapy could induce significant risks of GI bleeding, even with low-dose aspirin [3].

To overcome this issue, the concomitant use of a GI protective agent such as proton-pump inhibitors (PPIs) has been recommended for this patient population to attenuate the risk of GI bleeding [4]. A systematic review and meta-analysis demonstrated that PPI use was associated with a reduction in adverse GI events in patients treated with antiplatelet agents [5]. In addition, several observational and randomized studies have shown that PPIs are associated with a reduced risk of upper GI bleeding in patients who use antiplatelet agents [6,7,8,9]. Moreover, according to the key guidelines for reducing the GI risks of antiplatelet therapy and NSAID utilization, the concomitant use of PPIs and antiplatelet agents is recommended for patients with a history of GI bleeding to reduce recurrent bleeding complications [10]. These findings suggest that PPIs can be effectively used to prevent recurrent bleeding in patients with GI bleeding who use antiplatelet agents.

In terms of cardiovascular protection, there is some evidence for the possibility of an increased adverse cardiovascular event in patients treated with concomitant PPIs and antiplatelet therapy due to potential drug–drug interactions [11,12,13]. Additionally, a meta-analysis showed that although clopidogrel (an antiplatelet agent) alone appeared to be superior in reducing cardiovascular event risks, it may be associated with an increased risk of cardiovascular events when combined with PPIs [14]. Thus, using a combination of PPIs and antiplatelet agents is difficult to justify because GI protection may be achieved at the expense of cardiovascular event prevention. Therefore, this study aimed to evaluate the association of a dual therapy comprising antiplatelet agents and PPIs with adverse cardiovascular event risks using a nationwide population-based cohort dataset.

## 2. Materials and Methods

### 2.1. Study Dataset

Data from the Korean National Health Insurance Service National Sample Cohort (NHIS-NSC), a population-based cohort established by the Korean NHIS, were used in this study. Currently, the Korean NHIS maintains and stores records of healthcare utilization and prescriptions for the entire population as a single universal government insurer. South Korea has a single-payer national health system, which has covered the entire South Korean population since 1989. An insured individual pays for national health insurance, which is proportional to the individual’s income, and each South Korean is assigned a unique identification number at birth. With the integration of medical aid data into the NHIS database in 2006, this database comprises the entire population of South Korea. For these reasons, the claims data in the NHIS cannot be omitted or duplicated. Therefore, usage of the NHIS database eliminates selection bias. The NHIS-NSC is a representative sample cohort of 1,025,340 randomly selected participants, comprising 2.2% of the total eligible Korean population in 2002 who were followed up for 11 years until 2013. The NHIS-NSC was constructed using systematically stratified random sampling with 1476 strata in the context of age, sex, and income level. The cohort was refreshed annually by adding a representative sample of newborns sampled across 82 strata and removing subjects who were deceased or had emigrated using the 2.2% sampling rate during the follow-up period. The NHIS-NSC contains information about the participants’ insurance eligibility, medical treatment history, healthcare provider’s institution, and general health examination for each of the 12 years. Additionally, the reliability of the NHIS-NSC database has been validated, which showed a similar prevalence of 20 major diseases for each of the years assessed [15]. The present study adhered to the tenets of the Declaration of Helsinki and was approved by the Institutional Review Board (IRB) of Hallym Medical University, Chuncheon Sacred Hospital (No. 2021-08-006). The requirement for written informed consent was waived by the IRB because the NHIS-NSC database used in this study comprised de-identified secondary data. The authors confirm that data supporting the findings of this study are available within the article.

### 2.2. Study Setting and Participants

**Figure 1:**
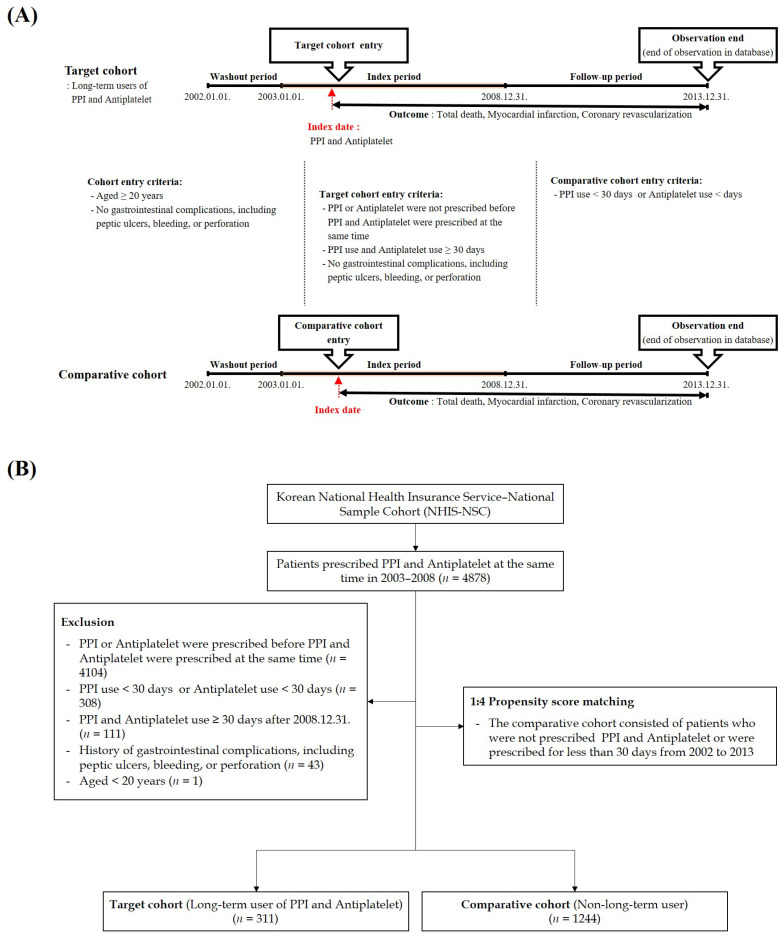
(**A**) Description of the study design. Target cohort: patients who had been prescribed concomitant antiplatelet agents and PPIs for 30 days during the index period and who were over 20 years of age. Comparative cohort: patients who had been prescribed antiplatelet agents without PPI usage during the index period and who were over 20 years of age. (**B**) Enrollment flow of the study participants.

This was a retrospective, nationwide propensity score-matched cohort study using the dataset from the National Health Claims Database collected by the NHIS. The datasets generated and/or analyzed in the present study are not publicly available because of the Korean National Health Insurance Service policies, but are available from the corresponding author upon reasonable request. In this study, all disease diagnostic codes were identified using the International Classification of Diseases, 10th revision (ICD-10). A schematic description of the study design and the flow of enrollment of the study participants is presented in Figure 1.

Briefly, to remove any potential pre-existing cases of primary outcomes, we excluded the first year (2002) from the dataset as the wash-out period. The patient group in this study included all those who had a prior history of acute coronary syndrome and had been prescribed the concomitant use of antiplatelet agents and PPIs during the index period (January 2003 to December 2008). The balanced operational definition of the concomitant use of PPIs and antiplatelet agents was those who had been prescribed PPIs and antiplatelet agents simultaneously for >30 days. In addition, we excluded the following patients: (1) those under 20 years of age; (2) those prescribed PPIs or antiplatelet agents before concomitant usage or concomitant usage < 30 days within the index period; and (3) those who experienced GI complications, including peptic ulcers, bleeding, or perforation before concomitant usage < 30 days. To select the comparison group, we randomly identified propensity score-matched participants (1:4) from the remaining cohort registered in the database as participants who were prescribed antiplatelet agents without the usage of PPIs during the entire period. Thus, participation in the comparison group consisted of those who had a prior history of acute coronary syndrome with prescribed antiplatelet agents. Patients who died within the index period were excluded from the comparison group. Therefore, the study cohorts consisted of subjects over 20 years of age who had been prescribed concomitant antiplatelet agents and PPIs for more than 30 days (target cohort) or without PPI usage (comparative cohort) during the index period. We included the following PPIs in the present study: esomeprazole, lansoprazole, and omeprazole. We defined the primary outcome event using the ICD code. The operational definitions of the study endpoints were all-cause mortality, myocardial infarction (diagnostic codes I21 and I22), and coronary revascularization (diagnostic codes M6551, M6552, M6553, M6554, M6561, M6562, M6563, M6564, M6565, M6566, M6567, M6571, M6572, M6634, and M6638). The risk of death, myocardial infarction, and coronary revascularization were compared between the two groups using person/years at risk, which was defined as the duration between the end of the concomitant usage of >30 days or 1 January 2009 (for comparison) and their respective endpoints.

### 2.3. Outcome Variables

In this study, we included the following covariates as independent variables: sex, age, residence, income level, and comorbidities. The study population was divided into three age categories (<45, 45–64, and >64 years), three residential areas (Seoul, the largest metropolitan region in South Korea; other metropolitan cities in South Korea (Busan, Daegu, Daejeon, Gwangju, Ulsan, and Incheon); and small cities and rural areas), and three income levels (≤30%, 30.1–69.9%, and ≥70% of the group median income). Using diagnostic codes, we also analyzed comorbidities, including essential hypertension (I10–I15), type 2 diabetes mellitus (E10–E14), and chronic kidney disease (N18). We defined the presence of comorbidities as any diagnosis during the index period prior to the prescription of antiplatelet agents combined with PPIs for more than 30 days. Patients who had experienced no events and were alive until 31 December 2013 were censored after this timepoint.

### 2.4. Statistical Analyses

Although 1:1 matching may yield sufficiently precise estimates in large studies or studies with strong effects, 1:n nearest neighbor matching is a reasonable way to improve the precision with little cost in bias [16]. Thus, we selected a 1:4 matching strategy, depending on the sizes of the exposed and comparison populations, to optimize the results. To identify whether patients treated with concomitant PPIs and antiplatelet agents after an acute coronary syndrome bleeding history had an increased risk of total death, myocardial infarction, and coronary revascularization, we used Cox proportional hazard regression analyses to calculate the hazard ratio (HR) and 95% confidence interval (CI), adjusted for the other independent variables. During the follow-up period, the Kaplan–Meier method was used to calculate the specific free time between the patient and the comparison groups. All statistical analyses were performed using R version 4.0.0, with a 2-tailed *p*-value significance level of 0.05.

## 3. Results

### 3.1. Demographic and Clinical Characteristics

**Figure 2:**
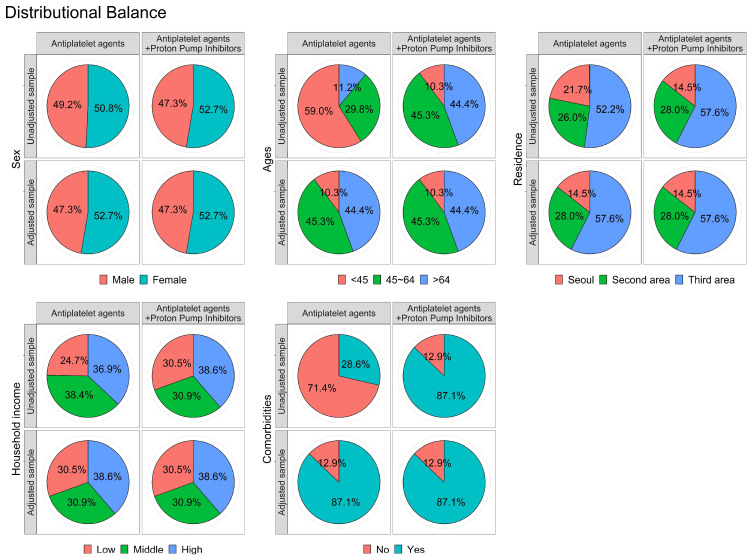
Balance plot for 5 variables before and after matching. The graph shows how the matching results changed so that the distribution of each independent variable was the same between the two groups.

In total, 1244 participants in the comparison group and 311 patients treated with concomitant PPIs and antiplatelet agents after a history of acute coronary syndrome were enrolled in this study. Table 1 presents patient characteristics, including sex, age, residence, household income, and comorbidities. The distributions of sex, age, residential area, household income, and comorbidities were similar between the groups. To confirm the effectiveness of propensity score matching, we analyzed the balance plot between the comparison and patient groups (Figure 2). All independent variables showed similar distributions between the two groups, indicating that each variable was appropriately matched.

### 3.2. Incidence and Risk Ratio

The incidence and risk ratios of total death, myocardial infarction, and coronary revascularization are presented in Table 2. The overall incidence of total death was 18.45 and 31.9 per 1000 person/years in the comparison and dual therapy groups, respectively. Additionally, we detected a higher incidence of myocardial infarction (4.46 per 1000 person/years) and coronary revascularization (6.74 per 1000 person/years) in the dual therapy group than in the control group (1.14 per 1000 person/years for myocardial infarction and 1.36 per 1000 person/years for coronary revascularization).

In the univariate and multivariate Cox regression analyses, we found that the adjusted HR for total death was significantly increased in the dual therapy group (adjusted HR = 1.77; 95% CI: 1.30–2.40). Additionally, incident myocardial infarction and coronary revascularization events were associated with an increased risk ratio in the dual therapy group compared with the comparison group (adjusted HR = 3.52; 95% CI: 1.34–9.22 and adjusted HR = 4.74; 95% CI: 2.03–11.05, respectively). The data for the time-to-event and censored events are shown in Table 3.

**Figure 3:**
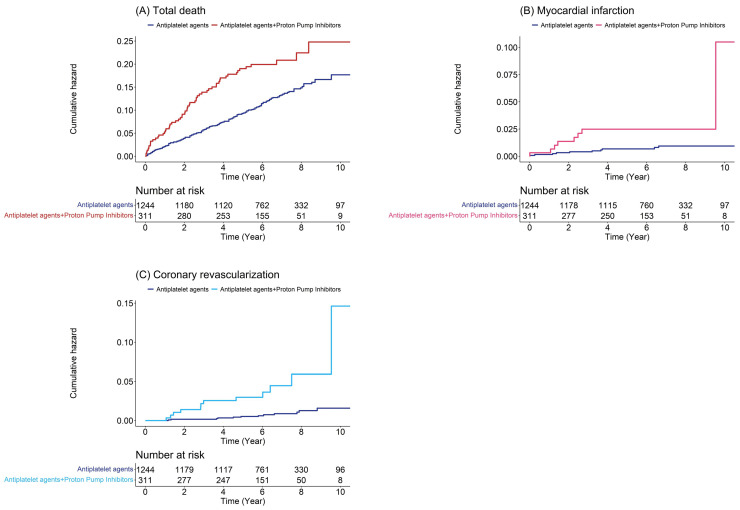
Cumulative hazard plot of specific events using the Kaplan–Meier method: (**A**) total death; (**B**) myocardial infarction; (**C**) coronary revascularization.

Kaplan–Meier survival curves with log-rank test results indicated that patients in the dual therapy group suffered from total death, myocardial infarction, and coronary revascularization more frequently than those in the comparison group (Figure 3).

Specifically, in the subgroup analysis, the highest risk of developing myocardial infarction and coronary revascularization events was observed in middle-aged patients treated with dual therapy compared with young or elderly patients treated with dual therapy (Table 4).

Moreover, we found that the adjusted HRs of myocardial infarction and coronary revascularization events were relatively higher within 3 years after the concomitant use of antiplatelet agents and PPIs, whereas they decreased then remained constant during the follow-up period (Table 5).

## 4. Discussion

Antiplatelet therapy, from low-dose aspirin to clopidogrel, is the most commonly used regimen for the management of patients with cardiovascular diseases. Generally, antiplatelet drugs are known to include antithrombotic agents as major components, which are mainly prescribed for the treatment and prevention of atherothrombotic diseases, including coronary artery disease, peripheral artery disease, ischemic strokes, and transient ischemic attacks [17,18,19,20]. Antiplatelet drugs exert their effects by either preventing the formation of second messengers, interacting with intracellular signaling pathways and blocking membrane receptors, or inhibiting platelet aggregation. However, prior studies have demonstrated the occurrence of varying degrees of bleeding complications with combination antiplatelet therapies [17,18,19,20]. Specifically, a GI hemorrhage is the most frequently reported bleeding complication associated with antiplatelet agents and it usually occurs in dose-dependent patterns [21,22]. To the best of our knowledge, this is the first study to compare the outcomes of total death, myocardial infarction, and coronary revascularization between patients administered dual therapy (antiplatelet agents with PPIs) and antiplatelet agents alone (without PPIs) in a national cohort of Asian patients discharged after hospitalization for acute coronary syndrome. This study, based on the South Korean NHIS-NSC, found that after adjustments for propensity scores, patients with a history of acute coronary syndrome who used concomitant dual therapy were at a higher risk of total death, myocardial infarction, and coronary revascularization events compared with non-PPI users. Additionally, we observed that middle-aged patients or those with 3 years of concomitant use showed a higher risk of myocardial infarction and coronary revascularization compared with the controls.

It is well known that clopidogrel is activated by the liver enzyme CYP2C19 and PPIs are metabolized by several human cytochromes such as P450, but only pantoprazole is metabolized by a sulfotransferase. Previous studies have demonstrated that the potential clopidogrel–PPI interaction is not likely to affect the entire class of PPIs because PPIs are metabolized by both CYP 450 2C19 and 3A4 in different proportions based on their isomeric forms [23,24,25]. Additionally, some studies suggest that the attenuating effects of concomitant PPI use on platelet response to clopidogrel are restricted to omeprazole [26,27,28]. Meanwhile, pantoprazole does not appear to completely inhibit CYP 450 2C19 and has not been linked to this adverse effect on clopidogrel [28].

To date, there is conflicting evidence regarding whether the use of concomitant PPIs and antiplatelet agents increases the risk of adverse cardiovascular events. Recent systematic reviews and meta-analyses suggest that, in terms of cardiovascular protection, clopidogrel alone appears to be superior to clopidogrel plus PPIs in reducing cardiovascular risk, whereas long-term mortality is not statistically significant [14,29]. However, these meta-analyses have critical limitations because they consist of observational studies and not randomized studies. Previous studies on healthcare claims data have also reported conflicting findings regarding the potential interaction between clopidogrel and PPIs. Similar to previous observational studies, a study based on Taiwan’s insurance database demonstrated that patients prescribed clopidogrel plus PPIs had a significantly higher incidence of cardiovascular events [30]. Additionally, another study based on Taiwan’s insurance database detected that the concomitant use of PPIs was associated with a significant reduction in risk among aspirin users but not among clopidogrel users [31]. In contrast, another study showed no apparent cardiovascular interaction between clopidogrel and PPIs in patients who received dual therapy [32].

Contrary to prior studies, we selected dual therapy subjects treated with antiplatelet agents and PPIs simultaneously for more than 30 days. Additionally, we identified control subjects prescribed antiplatelet agents without PPIs. Thus, we believe that our study design was appropriate to determine the precise effect of PPIs on drug–drug interactions in patients with acute coronary syndrome. However, to select concomitant users for more than 30 days, we could not divide the patients according to antiplatelet agents such as aspirin and clopidogrel. Thus, we found increased myocardial infarction and coronary revascularization events during the follow-up period, but we could not find any association between dual therapy and coronary artery disease, peripheral vascular disease, ischemic strokes, or transient ischemic attacks. Moreover, our research has other distinctive advantages compared with previous studies. First, this study minimized surveillance bias on the risk of total death and adverse cardiovascular events in patients treated with concomitant usage through the selection of sociographically matched controls in the cohort database. Second, although this study was designed retrospectively, it used a cohort dataset rather than a cross-sectional dataset. Thus, we investigated the risk ratio during the follow-up period and evaluated the change in risk over time. Third, we evaluated the risk ratio according to age category and time series during the follow-up period. This is important for clinicians because it might reveal an insight into which age group is more susceptible to the adverse effects of concomitant usage and the period in which patients treated with dual therapy are at a high risk of adverse cardiovascular events. Finally, although we combined all PPIs and antiplatelet agents into one category, we assessed concomitant users and their matching controls for more than 30 days. Thus, we evaluated the precise effect of dual therapy on cardiovascular protection due to drug–drug interactions.

However, our study had a few limitations. First, we could not access any specific personal health data such as smoking history and alcohol consumption. Therefore, we could not adjust for these confounding factors. Second, the diagnosis of the disease was dependent only on ICD-10 diagnostic codes, which may be less accurate than diagnoses based on medical chart data as these often include the medical history, physical examinations, and laboratory results. Consequently, this study had the potential for misclassification bias. Third, the NHIS-NSC database provides categorized age data (<45, 45–64, and >64 years), which is why we could not match the two groups according to the actual age distribution; our findings may have some residual bias within the categories as a result. Fourth, this study could not present a direct association of dual therapy with total death or adverse cardiovascular events due to our study design, in which the baseline characteristics of the individuals were limited to a previous database. Therefore, we could not confirm whether our findings had a causal relationship or a temporal incidence. Finally, in this study, the exact medication compliance was unclear, although we considered the selection of concomitant prescriptions. Further studies are required to confirm these issues.

## 5. Conclusions

We investigated the possible effect of the concomitant use of PPIs and antiplatelet agents on the risk of adverse cardiovascular events. The present study revealed that patients treated with dual therapy had a significantly higher risk ratio of total death, myocardial infarction, and coronary revascularization than non-PPI users. Additionally, middle-aged patients treated with dual therapy showed an increased risk of total death, myocardial infarction, and coronary revascularization compared with young or elderly patients. Therefore, clinicians must be aware of the potential risk of adverse cardiovascular events when prescribing dual therapy to patients with acute coronary syndrome.

## Figures and Tables

**Table 1:** Characteristics of the study participants.

Variables	Antiplatelet Agents (Clopidogrel) (*n* = 1244)	Antiplatelet Agents and Proton-Pump Inhibitors (*n* = 311)	*p*-Value
Sex			1.000
Male	588 (47.3%)	147 (47.3%)	
Female	656 (52.7%)	164 (52.7%)	
Ages (years)			1.000
<45	128 (10.3%)	32 (10.3%)	
45–64	564 (45.3%)	141 (45.3%)	
>64	552 (44.4%)	138 (44.4%)	
Residence			1.000
Seoul	180 (14.5%)	45 (14.5%)	
Second area	348 (28.0%)	87 (28.0%)	
Third area	716 (57.6%)	179 (57.6%)	
Household income			1.000
Low (0–30%)	380 (30.5%)	95 (30.5%)	
Mid (30–70%)	384 (30.9%)	96 (30.9%)	
High (70–100%)	480 (38.6%)	120 (38.6%)	
Comorbidities			1.000
No	160 (12.9%)	40 (12.9%)	
Yes	1084 (87.1%)	271 (87.1%)	
Hypertension			0.106
No	347 (27.9%)	72 (23.2%)	
Yes	897 (72.1%)	239 (76.8%)	
Type 2 diabetes			0.005
No	794 (63.8%)	171 (55.0%)	
Yes	450 (36.2%)	140 (45.0%)	
Chronic kidney disease			<0.001
No	1212 (97.4%)	288 (92.6%)	
Yes	32 (2.6%)	23 (7.4%)	

Comparison: subjects matched to the dual therapy group; Seoul: the largest metropolitan area; second area: other metropolitan cities; third area: other areas in South Korea.

**Table 2:** Comparison of the incidence per 1000 person/years and the risk ratio of primary outcomes between the comparison group and patients treated with dual therapy (proton-pump inhibitors and antiplatelet agents).

Variables	*n*	Case	Person/Year	Incidence	Unadjusted HR (95% CI)	Adjusted HR (95% CI)	*p*-Value
Total Death							
Antiplatelet agent (clopidogrel)	1244	163	8820.1	18.48	1.00 (ref)	1.00 (ref)	
Antiplatelet agents and proton-pump inhibitors	311	58	1814.1	31.97	1.68 (1.25–2.28) ***	1.77 (1.30–2.40) ***	<0.001
Myocardial Infarction							
Antiplatelet agent (clopidogrel)	1244	10	8798.9	1.14	1.00 (ref)	1.00 (ref)	
Antiplatelet agents and proton-pump inhibitors	311	8	1795.6	4.46	3.77 (1.47–9.63) **	3.52 (1.34–9.22) *	0.010
Coronary Revascularization							
Antiplatelet agent (clopidogrel)	1244	12	8798.1	1.36	1.00 (ref)	1.00 (ref)	
Antiplatelet agents and proton-pump inhibitors	311	12	1780.7	6.74	5.53 (2.45–12.45) ***	4.74 (2.03–11.05) ***	<0.001

Comparison: subjects matched to the dual therapy group; HR: hazard ratio; CI: confidence interval. * *p* < 0.05; ** *p* < 0.010; *** *p* < 0.001.

**Table 3:** The numbers of each final finding during the follow-up period between the comparison group and patients treated with dual therapy (proton-pump inhibitors and antiplatelet agents).

	Total Deaths	Myocardial Infarction	Coronary Revascularization
Event	221	18	24
Antiplatelet agent (clopidogrel)	163	10	12
Antiplatelet agents and proton-pump inhibitors	58	8	12
Total censored (no event)	1334	1537	1531
Antiplatelet agent (clopidogrel)	1081	1234	1232
Antiplatelet agents and proton-pump inhibitors	253	303	299
Termination of study	1270	1260	1248
Antiplatelet agent (clopidogrel)	1031	1025	1020
Antiplatelet agents and proton-pump inhibitors	239	235	228
Loss to follow-up/drop-out	64	277	283
Antiplatelet agent (clopidogrel)	50	209	212
Antiplatelet agents and proton-pump inhibitors	14	68	71

Comparison: subjects matched to the dual therapy group.

**Table 4:** Hazard ratios of primary outcomes between the comparison group and patients treated with dual therapy (proton-pump inhibitors and antiplatelet agents) according to age group.

Ages	<45		45–64		>64	
	Antiplatelet Agent (Clopidogrel)	Antiplatelet Agents and Proton-Pump Inhibitors	Antiplatelet Agent (Clopidogrel)	Antiplatelet Agents and Proton-Pump Inhibitors	Antiplatelet Agent (Clopidogrel)	Antiplatelet Agents and Proton-Pump Inhibitors
Total Deaths						
Unadjusted HR (95% CI)	1.00 (ref)	-	1.00 (ref)	2.28 (1.10–4.70) *	1.00 (ref)	1.75 (1.25–2.44) **
Adjusted HR (95% CI)	1.00 (ref)	-	1.00 (ref)	2.08 (0.97–4.46)	1.00 (ref)	1.77 (1.26–2.49) ***
Myocardial Infarction						
Unadjusted HR (95% CI)	1.00 (ref)	-	1.00 (ref)	25.01 (2.64–237.30) **	1.00 (ref)	2.02 (1.62–6.57) *
Adjusted HR (95% CI)	1.00 (ref)	-	1.00 (ref)	16.79 (1.81–156.10) *	1.00 (ref)	2.09 (1.63–6.89) *
Coronary Revascularization						
Unadjusted HR (95% CI)	1.00 (ref)	-	1.00 (ref)	17.12 (4.52–64.88) ***	1.00 (ref)	1.95 (1.52–7.31) *
Adjusted HR (95% CI)	1.00 (ref)	-	1.00 (ref)	13.64 (3.41–54.51) ***	1.00 (ref)	1.74 (1.45–6.72) *

Comparison: subjects matched to the dual therapy group; HR: hazard ratio; CI: confidence interval. * *p* < 0.05; ** *p* < 0.010; *** *p* < 0.001.

**Table 5:** Risk of myocardial infarction or coronary revascularization event by time elapsed since the concomitant usage of proton-pump inhibitors and antiplatelet agents.

Time (Year)	Myocardial Infarction	Coronary Revascularization
	Adjusted HR (95% CI)	Adjusted HR (95% CI)
1	3.56 (0.32–39.47)	No events
2	4.16 (0.98–17.63)	9.44 (1.51–59.03) *
3	6.02 (1.86–19.51) **	15.33 (3.08–76.34) ***
4	3.69 (1.31–10.43) *	7.35 (2.07–26.07) **
5	3.69 (1.31–10.43) *	5.54 (1.87–16.36) **
6	3.69 (1.31–10.43) *	4.45 (1.55–12.76) **
7	3.17 (1.18–8.52) *	4.65 (1.83–11.87) **
8	3.17 (1.18–8.52) *	4.61 (1.93–11.01) ***
9	3.17 (1.18–8.52) *	4.46 (1.90–10.50) ***
10	3.52 (1.34–9.22) *	4.74 (2.03–11.05) ***
11	3.52 (1.34–9.22) *	4.74 (2.03–11.05) ***

HR: hazard ratio; CI: confidence interval. * *p* < 0.05; ** *p* < 0.010; *** *p* < 0.001.

## Data Availability

The authors confirm that the data supporting the findings of this study are available within the article.
